# Environmental Evaluation of Yellow Mealworm Larvae Products: Analysis of Modeling Choices and Nutritional Impact-Adjusted Comparison

**DOI:** 10.1021/acsomega.5c03159

**Published:** 2026-02-06

**Authors:** Ana Fernández-Ríos, Jara Laso, Rubén Aldaco, María Margallo

**Affiliations:** Department of Chemical and Biomolecular Engineering, 16761 University of Cantabria. Av. de los Castros s/n, Santander 39005, Spain

## Abstract

The transition of eating habits to meet safe nutritional recommendations, coupled with the strong environmental interactions of current animal production systems, boosts the development of this research. This study conducts the life cycle assessment (LCA) and nutritional LCA (n-LCA) of *Tenebrio molitor* larvae and a derived food product (lasagna). Both attributional with economic allocation (aLCA) and consequential (cLCA) modeling approaches were employed as baseline scenarios to address the multifunctionality, complemented by a sensitivity analysis that evaluates alternative allocation strategies. The aLCA results identified insect feed production as the bottleneck in the larval production system, while the other ingredients were the primary environmental hotspot in the lasagna system. The choice of the modeling approach influenced the results: the cLCA scenario showed slightly higher impacts for mealworm production (e.g., 1.49 kg CO_2_ equiv/kg) compared to the aLCA baseline (1.45) and aLCA with mass allocation (0.66), but lower than for aLCA with no allocation (1.80). In contrast, the lasagna system benefited from the cLCA approach, with 5.25 CO_2_ equiv/meal, while the aLCA scenario reported 5.40 kg CO_2_ equiv. Finally, although the nutritional quality of *T. molitor* was relatively low compared to other protein-rich foods, its nutritionally adjusted environmental impacts were generally lower than those of conventional animal products. This highlights both the challenges and the potential opportunities for the development of insect-based food systems.

## Graphical abstract

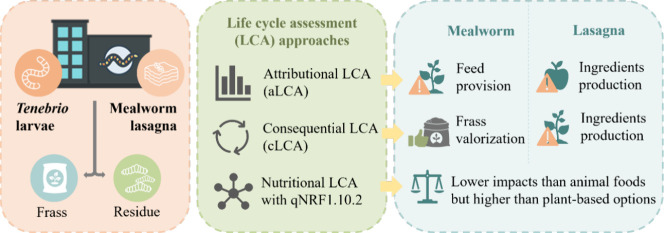

## 1 Introduction

Proteins are responsible for nearly every task of cellular life and serve as structural support, biochemical catalysts, hormones, enzymes, and building blocks of bones, skin, organs, and muscles. To meet the functional needs, protein intake differs between individuals depending on their age, gender, health status, or physical activity levels, with 0.8 g/kg body weight as the recommended intake and consumption up to 2 g/kg body weight as a safe (tolerable) limit.^1^ However, there is an underlying issue with the patterns of protein consumption and its distribution. On the one hand, Moughan^2^ demonstrated that, even though the average daily per capita gross protein intake is met for an extensive sample of regions, when considering the protein digestibility and utilization, the average consumption is below the required level, which could have potentially serious implications. On the other hand, proteins from plant-based products are generally scarce in human diets, while consumption of animal proteins, particularly of red meat, exceeds recommendations.^3^ In fact, the daily supply of protein from animal products has increased by 90% from 1961 to 2021. Even though globally the intake of vegetal protein surpasses that of animal, in some regions such as Europe or Central America, this trend has been reversed in the late 1970s and early 2000s, respectively.^4^ Driven by the potential health side effects of excessive meat intake, coupled with the necessary change in eating patterns to meet recommendations and the strong environmental interactions of livestock systems, proteins from alternative sources are attracting increasing attention.^5^

One of the benefits of alternative proteins (APs) has been identified as health promotion, since products containing APs report lower energy density, cholesterol, and saturated fat but higher dietary fiber concentration and availability of micronutrients.^6^ In particular, insects are used as a food ingredient to increase the nutritional quality of meals around the world, with specific purposes such as the improvement of population health in countries where access to a protein source is scarce or the development of exotic products that complement and enhance unbalanced diets.^7^ Although entomophagy, i.e., insect consumption, is common in some Asian, African, or Latin American cultures, it is still unfamiliar to most Western populations.^8^ In European countries, insects are classified as ‘novel foods’, which have not been consumed to a significant degree by humans before 15 May 1997.^9^ The European Commission is currently working on the authorization of insects for human consumption, having already approved *Tenebrio molitor*, *Locusta migratoria*, *Acheta domesticus*, and *Alphitobius diaperinus*.^10^ The available evidence suggests protein concentration of edible insects similar to that of beef or pork, and most species meet the amino acid content recommended by the World Health Organization.^11^ Insects also appear to be a promising source of fiber, since their exoskeleton is composed of chitin, as well as of omega-3 polyunsaturated fatty acids, whose concentration could be comparable to that of fish and seafood.^12^ The presence of micronutrients, including iron, zinc, potassium, magnesium, or B vitamins, is outstanding and makes insects a powerful and regionally adaptable source of nutrition to help ameliorate food insecurity.^13^ In addition to their nutritional importance, insects have the potential to act as a more environmentally sustainable nutrient source than other widely consumed animal products.^14^ Even though environmental implications are determined by species, production method, and applied feed,^15^ major benefits are related to the relatively high feed conversion ratio (FCR), low water and land requirements, and small generation of greenhouse gases (GHG).^16^

Within this background, this contribution is focused on *Tenebrio molitor* (yellow mealworm), one of the first insects approved as safe to consume in the EU,^17^ and its potential as a healthy and sustainable food source. To date, numerous research studies have analyzed the nutritional quality of yellow mealworm, and some have conducted life cycle assessment (LCA) to estimate the environmental impacts linked to its production. Due to the recently widespread interest in this product, the first LCA dates back to 2018, which evaluates the environmental implications of *Tenebrio molitor* for feed purposes.^18^ More recently, Zlaugotne and colleagues^19^ complement this study by comparing the impacts with those of other protein sources, including black soldier fly or soybean protein. Dreyer and collaborators^20^ change the focus to human consumption, considering data from a farm located in Austria, and Tello et al.^21^ go a step further by evaluating the impacts of *Tenebrio molitor*-based milk. Finally, Laroche and colleagues^22^ calculate the carbon footprint and eco-efficiency of protein extract from *Tenebrio* using different extraction and purification methods. With the objective of contributing to the current state of the art, the present study applies LCA to analyze the environmental impacts and hotspots of a yellow mealworm farm and a hypothetical derivative product, namely, mealworm-based lasagna. In contrast to the existing literature, this paper studies the influence of modeling decisions on the results, assessing different attributional and consequential LCA scenarios to address the multifunctionality of the product system. While attributional LCA attempts to provide information on what portion of global environmental impacts can be associated with a specific product, consequential LCA aims to provide information on the burdens that occur, directly or indirectly, as a consequence of a decision, usually represented by changes in demand for a product.^23^ Besides, a comparative nutritional LCA is conducted to contrast the environmental profile in terms of the nutritional quality of *Tenebrio molitor* and other APs, along with the implications of their substitution in the meal. To do so, the quality nutrient-rich food 1.10.2 model (qNRF1.10.2), which combines nutrient intake adequacy and protein quality in a single score, is applied to measure the real function of the products.^24^ Based on the review, all publications address a nutritional LCA approach by considering the protein quantity, but fail in considering a more comprehensive perspective of nutrient density. In addition, they do not rely on different approaches, such as consequential LCA, to study the influence on environmental outcomes as a consequence of changes in the demand for products. This consequentialist idea that actors should be responsible for the consequences of their production and consumption actions is fundamental to environmental management systems.^25^ Therefore, a cLCA allows assessing all the causal-effect relations within the market by changing product demand using marginal data.^26^ In the particular case of insects, this has been identified as a necessity by Thévenot et al.^18^ for determining future directions of this sector.

## 2 LCA Methodology

### 2.1 Objective of the Study

The analysis was divided into two stages based on the expected products. First, LCA was applied to *Tenebrio molitor* larvae, with the objective of estimating the environmental impacts and identifying bottlenecks associated with the rearing and processing. Second, LCA was conducted for a ready-to-eat meal (lasagna), in which the protein source was replaced by yellow mealworm and other APs in order to evaluate and compare the environmental and nutritional impacts. An attributional (aLCA) approach was initially adopted in this study to evaluate the performance of a *status quo* scenario based on average real data, which constituted the baseline scenario and facilitated the clarification of the environmental hotspots of production. Due to the multifunctionality of the product system, in which frass is generated as the main byproduct, two scenarios composed the sensitivity analysis based on the definition of different attributional allocation procedures, which are explained in detail in Section 2.4. Additionally, a consequential LCA (cLCA) perspective was performed to assess the influence of the approach change on environmental results. cLCA attempts to provide information on the direct and indirect environmental burdens as a aconsequence of a decision.^27^ In this case, the implications of an increase in the demand for yellow mealworm products were studied.

### 2.2 Function and Functional Units (FU)

At this point, it is essential to differentiate between the functions of the product system and the functions of the final products. The primary function of the system was to produce yellow mealworm and subsequently a meal intended for food purposes, while the function of these products was to provide nutrients and bioactive compounds for the sustenance and development of the human body.

Given that it is a multifunctional process, four mass-based FUs that reflect the different functions of the products obtained were used in aLCA: (i) 1 kg of yellow mealworm larvae ready for human consumption, (ii) one lasagna with the protein source (beef) replaced by *Tenebrio molitor*, (iii) 1 kg of frass generated as coproduct of mealworm rearing, and (iv) 1 kg of mealworm residues. Additionally, to represent the real function of the desirable products, the quality nutrient-rich food 1.10.2 scores of *Tenebrio molitor* and of the meals were applied as FUs. This nutrient profiling model measures the adequacy of macro- and micronutrient intake and introduces the digestible indispensable amino acid score (DIAAS) to provide the protein content adjusted to its digestibility and bioavailability.^24^ The algorithm for the estimation of the qNRF1.10.2 scores is presented in eq 1.

$$\mathrm{qNRF}1.10.2=\left(\frac{\mathrm{protein}\times \mathrm{DIAAS}}{{\mathrm{DRI}}_{p}}+\sum_{i=10}\left(\frac{{\mathrm{nutrient}}_{i}}{{\mathrm{DRI}}_{i}}\right)-\sum_{j=2}\left(\frac{L_{j}}{{\mathrm{MRI}}_{j}}\right)\right)$$ 1

where protein represents the protein content in a food expressed in g/100g, DIAAS is the digestible indispensable amino acid score (%), DRI_p_ is the daily recommended intake for protein, nutrient_*i*_ is the concentration of nutrient “*i*” to encourage (*i* = fiber, vitamins A, B9, B12, E, D, Zn, Mg, Ca, and Fe) in 100 g of food, DRI_*i*_ is the daily recommended intake for nutrient “*i*”, *L*_*j*_ is the content of disqualifying nutrient “*j*” (*j* = saturated fatty acids and Na) in 100 g of food, and MRI_*j*_ is the daily maximum recommended intake of nutrient “*j*”.

For the consequential analysis, FUs of increase of market demand for (i) 1 kg of dried *Tenebrio molitor* larvae and (ii) 1 mealworm-based meal (lasagna) were defined.

### 2.3 System Boundaries and Description

System boundaries were set from cradle to consumer, which includes raw materials production and supply, mealworm rearing, killing and blenching, maintenance of facilities, intermediate waste management, production of the final meal, and dehydration of mealworm manure and residues to produce commercial frass and feed (Figure 1). Transportation from industry to households was dismissed due to a lack of reliable data and to the expected low contribution of this stage to the overall impacts since the company only commercializes the products at the national level. Although most food-related LCA studies adopt a cradle to gate approach, for nutritional LCA is preferable for system boundaries to extend to the consumption stage to consider changes in nutrient characteristics and their bioavailability after home storage and cooking.^28^

**Figure 1:**
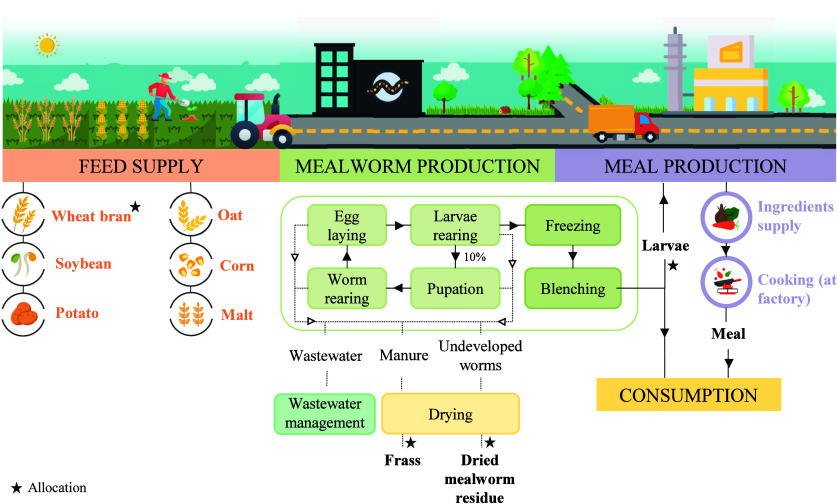
Flow diagram of the system under study.

Regarding *Tenebrio molitor* production, soil preparation, cultivation, fertilization, harvesting, and other on-field activities concerning the production of feed ingredients were included within the limits. Besides, transportation of feed, both from wholesaler to supplier and from supplier to the farm, was considered. The farm under study was located in Pontevedra (NW Spain) and had an annual production capacity of 18 tons of *Tenebrio molitor* larvae (live weight). Mealworm rearing was performed in a climate-controlled chamber, with a temperature of 26 °C and humidity levels around 65%. The annual production cycle comprised 12 months during which three litters were reared. This operation was carried out in plastic trays, which contain feeding substrate composed of cereals, including wheat bran, oat, corn, and soybean. Additionally, wet feed consisting of potato and malt was fed every 2 days as a nutritional supplement. Cleaning agents for vegetables were included in the system. The feed conversion ratio (FCR), i.e., the amount of feed ingested to produce 1 kg of larvae, was estimated at 4. The rearing process started with egg laying and larval development. From week 14 onward, 90% of the larvae produced were extracted for subsequent stages, whereas the remaining 10% were maintained in the trays for reproduction (pupation and insect rearing). The maintenance of the facilities and cleaning of the rearing trays were also included within the system boundaries. The former was carried out by daily cleaning of the chamber using chemical products (floor cleaners) and antimite to avoid contamination of external agents. Tray cleaning was performed by removing fresh feed residues, undeveloped dead mealworms, and excess manure from the trays, leaving a thin layer that acts as thermal insulation. The organic waste (1–2 kg per day) is deposited in containers for its management, while the undeveloped mealworms (average 11 kg per week) were dehydrated in a dryer for 8 h and sold to a birds’ feed factory. On the other hand, surplus manure was also dehydrated and sold to be utilized as organic fertilizer. This frass had an NPK content of 3/3/3, a concentration of organic matter of 80% and a pH of 6.5. Turning to the life cycle of the larvae, once collected, they were subjected to freezing for killing and blenching for elimination of potential pathogens.^20^ These stages were considered according to the literature, as at the time of data collection in the industry under study, the use of insects for human consumption was starting in Spain, and therefore, live insects were sold for use as animal feed.

The system addressing the production of lasagna was hypothetical and considered the production of all ingredients and the cooking process. This system was created from a homemade recipe, taking the quantities of each ingredient and following all the steps for its preparation. For an update of the traditional meal containing beef, a substitution with mealworm or other AP was conducted using the qNRF1.10.2 scores. Based on this, the amount of mealworm should be 1.25 times the quantity of beef to provide the same nutritional quality. Like in the previous “subsystem”, cereals and vegetables cultivation and processing were included within the boundaries, along with the production of dairy products and fats such as oils. Transportation of these ingredients was not considered in the system due to a lack of real and reliable data. The cooking process included the consumption of water and the use of an electric stove for pasta boiling, saute, meat, and white sauce preparation, as well as the utilization of an oven for final grilling.

### 2.4 Multifunctionality in Attributional Scenarios

As previously mentioned, the production of the desirable product, i.e., *Tenebrio molitor* larvae, entails the generation of coproducts, namely, frass and mealworm residue consisting of dead and undeveloped worms (Figure 1). This multifunctionality was solved in the attributional model by addressing economic allocation, as did Tello et al.^21^ This scenario will be referred to as ‘TM_AE_’ throughout the manuscript. To do so, an average price of 2.44 EUR/100 g of frass was estimated based on information on the company under study. The same price was considered for the dried mealworm residue, as it is subjected to the same operations as manure for its valorization. For insects, 22.10 EUR/100g was taken based on the average prices of the European market for insects.^29^ Therefore, 21.58% of the impacts were assigned to frass, 0.27% to dried mealworm residue, and 78.15% to mealworm larvae. On the other hand, a multifunctional process was also found in wheat bran production, which produces both bran and flour. In this case, economic allocation was used considering a market price of 0.64 EUR/kg of flour and 0.18 EUR/kg of bran, resulting in 89.37% of the impacts allocated to the flour and 10.63% to the bran. The use of economic allocation was justified as it aligns with the ISO allocation hierarchy, which prioritizes economic value when physical relationships are not adequate and ensures methodological consistency with the background databases applied.

According to the UNE-EN ISO 14044,^30^ when multifunctionality can be dealt with by different procedures, a sensitivity analysis should be performed to illustrate the consequences of moving away from the selected approach. Therefore, two changes in allocation were performed in the attributional model:

1. Economic allocation was avoided, and impacts were fully allocated to mealworms (scenario “TM_AW_”), since the production system was specifically designed for rearing and processing this product. This strategy has been adopted in other comparable LCA studies^20^ and would allow for a proper comparison of results.
2. Mass-based allocation was applied (“TM_AM_”), in which 70.78% of the impacts were allocated to frass, 0.90% to residue, and 28.32% to mealworm larvae. Results of this assessment are provided together with those of the base case in Section 3.2. Quantification of environmental impacts in the status quo (aLCA).

### 2.5 Multifunctionality under a Consequential Approach

For measuring environmental consequences associated with changes in the demand for the products, the adoption of a consequential approach (scenario “TM_C_”) was assumed. To do so, the expansion method was adopted to account for the broader environmental impacts that result from markets’ responses to changing product demand.

The marginal suppliers, i.e., producers that will change production capacity in response to a change in demand for the products (mealworm larvae and mealworm-based lasagna), were identified and included within the system boundaries. A growing demand for *Tenebrio molitor* products (nonconstrained market) will lead to an increase in mealworm production, for which feed is needed. Consequently, it will result in a response in the marginal market for cereals and tubers production to meet both human and mealworm demand. Coproducts were expected to meet their function. Dried mealworm residue will be used as insect-based feed for birds, thus reducing the demand for conventional feed. This market displacement was considered in calculations based on the adjusted protein content. Similarly, frass will be used as fertilizer, which will decrease the demand for other organic fertilizers. In this case, the organic matter concentration was taken into account, assuming a commercial organic fertilizer with an NPK content of 3/2/3 and an organic matter concentration of 52.7%.^31^ Figure 2 illustrates the diagram flow with the counterfactual units.

**Figure 2:**
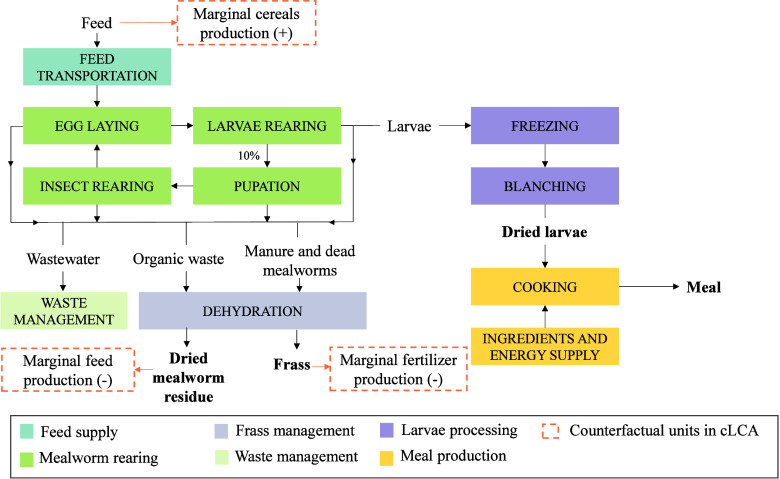
Flow diagram of consequential LCA modeling.

### 2.6 Life Cycle Inventory (LCI)

LCI data for *Tenebrio molitor* production were provided by a mealworm production industry located in NW Spain. Data compilation was performed by questionnaires and interviews with the managers, taking information from the year 2022. Information included a description of the production system, quantities and supply of the resources for rearing and processing yellow mealworm: feed ingredients, electricity for the climatic chamber and dryers, water, or cleaning agents, among other materials. For modeling in the LCA software, background processes from the Ecoinvent v3.10 database—using the cutoff system model for the attributional scenarios and the consequential system model for the consequential scenario—and from the Agribalyse 3.1.1. database were used. The limitations related to the selection of these databases are discussed in the conclusions section. A summary of the LCI is provided in Table 1, and background processes and details for the adaptation can be consulted in Tables S.1 and S.2 of the Supporting Information.

**Table 1:** Life Cycle Inventory for the Production of 1 kg of *Tenebrio molitor* Larvae

input/output	unit	quantity	data source
land	[m^2^]	7.77 × 10^–2^	producer
wheat bran	[kg]	2	producer
oat	[kg]	0.43	producer
corn	[kg]	0.62	producer
soybean	[kg]	6.76 × 10^–2^	producer
potato	[kg]	0.71	producer
transport – distance for feed ingredients, except barley (wholesaler-supplier)	[km]	227	producer
transport – distance for feed ingredients, except barley (supplier-producer)	[km]	5	producer
barley	[kg]	0.15	producer
transport – distance for barley (wholesaler-supplier-producer)	[km]	30	producer
electricity, for climatic chamber	[kWh]	1.08 × 10^–2^	own calculations based on producer data
rearing boxes (PP)	[kg]	4 × 10^–3^	^20^
water, for feed cleaning	[L]	0.30	producer
water, for facilities maintenance	[L]	0.10	producer
bleach	[L]	1.11 × 10^–3^	producer
floor cleaner	[L]	5.55 × 10^–4^	producer
antimite	[L]	2.77 × 10^–4^	producer
electricity, for killing	[kWh]	6.11 × 10^–2^	^20^
electricity, for blanching	[kWh]	0.46	^20^
electricity, for manure and residue low-temperature drying	[kWh]	1.20 × 10^–3^	own calculations based on producer data
N_2_O emissions	[kg]	2.55 × 10^–5^	^36^
CO_2_ emissions	[kg]	7.58 × 10^–3^	^36^
NH_3_ emissions	[kg]	1 × 10^–6^	^36^
yellow mealworm larvae	[kg]	1	
mealworm residues (dead, undeveloped)	[kg]	3.18 × 10^–2^	own calculations based on producer data
mealworm frass	[kg]	2.50	producer
residues of dried feed	[kg]	3.04 × 10^–2^	own calculations based on producer data
residues of wet feed	[kg]	1.01 × 10^–2^	own calculations based on producer data
wastewater	[L]	0.40	own calculations based on producer data

Details of the cultivation characteristics of cereals used as feeds were not provided, but it is known that all ingredients originate in Spain. Hence, LCIs from crops produced in Spain were considered from both the literature and LCA databases, and transportations were included based on the managers’ data. Adaptations of LCIs, e.g., update of electricity sources, regionalization of resources, etc., were performed to get the most realistic conditions (Tables S.1 and S.2). Transportation was considered to be carried out by small diesel lorries. Residues generated from nonconsumed dried and wet feed that are disposed of were considered to be treated according to the waste management strategies in Spain. Therefore, 5.67% of the organic waste was recycled, 22.90% composted, 58.15% landfilled, and 13.28% incinerated.^32^ Data concerning the rearing process were collected over a 12-month production period. Polypropylene was assumed as the material for the rearing boxes, for which a useful lifetime of 7.5 years was considered.^20^ Rearing emissions included N_2_O and NH_3_, and were estimated according to the measurements provided by Oonicx et al.^36^ CO_2_ emissions resulting from feed and substrate degradations were also considered in the analysis. Values reported by Oonicx et al.^33^ were taken as a proxy due to the similar composition of feed ingredients and rearing conditions. Regarding the cleaning agents, the antimite solution was modeled according to the chemical composition of the commercial product, composed of pyrethrin and rapeseed oil in a concentration of 4.59 and 823.3 g/L, respectively. In relation to the energy consumption, residual electricity was used. It consists of electricity that remains in the grid mix once the demand for renewable sources has been met. Therefore, since the producers use electricity from the Spanish grid mix, this percentage of green energy that has already been supplied to other consumers should be subtracted to avoid double-counting in the electricity supply. This resource was composed of approximately 58% of fossil resources, mainly natural gas and hard coal, 36% of nuclear, and 6% of renewable energies, especially wind and solar.^34^ The electricity requirements for the killing and blenching of the mealworm were estimated by Dreyer et al.^20^

The recipe and cooking procedure for lasagna meal were extracted from BBC^35^ and Schmidt Rivera and Azapagic.^36^ Ingredients and electricity were assumed to be produced in Spain whenever possible or in the average European markets. Electricity usage was estimated based on the average consumption of a stove and an oven, considering the cooking times specified in the recipe for each appliance. All inputs and outputs (LCI) for the meal production are reported in Table 2.

**Table 2:** Life Cycle Inventory for the Production of Lasagna Meal

input/output	unit	quantity
olive oil	[kg]	2.83 × 10^–2^
yellow mealworm mince	[kg]	1.12
onion	[kg]	0.22
garlic cloves	[kg]	2 × 10^–2^
wheat flour	[kg]	2.83 × 10^–2^
meat stock	[L]	0.15
tomato puree	[kg]	4.25 × 10^–2^
thyme	[kg]	1.41 × 10^–2^
chopped tomatoes	[kg]	0.80
butter	[kg]	5 × 10^–2^
wheat flour	[kg]	5 × 10^–2^
milk	[L]	0.75
mustard	[kg]	1 × 10^–2^
parmesan cheese	[kg]	5 × 10^–2^
lasagna sheets	[kg]	0.78
cheddar cheese	[kg]	7.50 × 10^–2^
electricity, for stove	[kWh]	1.80
electricity, for oven	[kWh]	0.90
tap water	[L]	5
lasagna	[unit]	1

### 2.7 Selection of Impact Categories

The SimaPro software was applied for modeling the product systems. Six midpoint impact categories comprised in the Product Environmental Footprint Category Rules were selected for their importance in the environmental assessment of the product systems: global warming potential (GWP), freshwater (FEP) and marine (MEP) eutrophication potential, land use (LU), water scarcity (WU), and fossil resources consumption (ADP fossil). The former and the latter were chosen due to their ability to measure the impacts of energy, as well as the emissions generated for *Tenebrio* rearing in the case of GWP. The eutrophication potential was considered relevant due to the role of mealworm production in the agricultural sector and the use of fertilizers for the production of feed ingredients. Finally, land and water use were measured as they provide a good framework for comparison with other protein sources, such as meat, which are very demanding of these resources. The Environmental Footprint 3.1 method was used in SimaPro v9.6 software. Additionally, environmental footprints on other impact categories are reported in Tables S.3 and S.4 of the Supporting Information. An uncertainty analysis was performed by using the Monte Carlo simulation, running a total of 1000 iterations and setting a confidence interval of 95%.

## 3 Results and Discussion

### 3.1 Identification of Environmental Hotspots

Figure 3 shows the relative contributions of the life cycle stages to the overall environmental impacts of *Tenebrio molitor* larvae (inner circle) and a lasagna meal with a mealworm as the protein source (outer circle).

**Figure 3:**
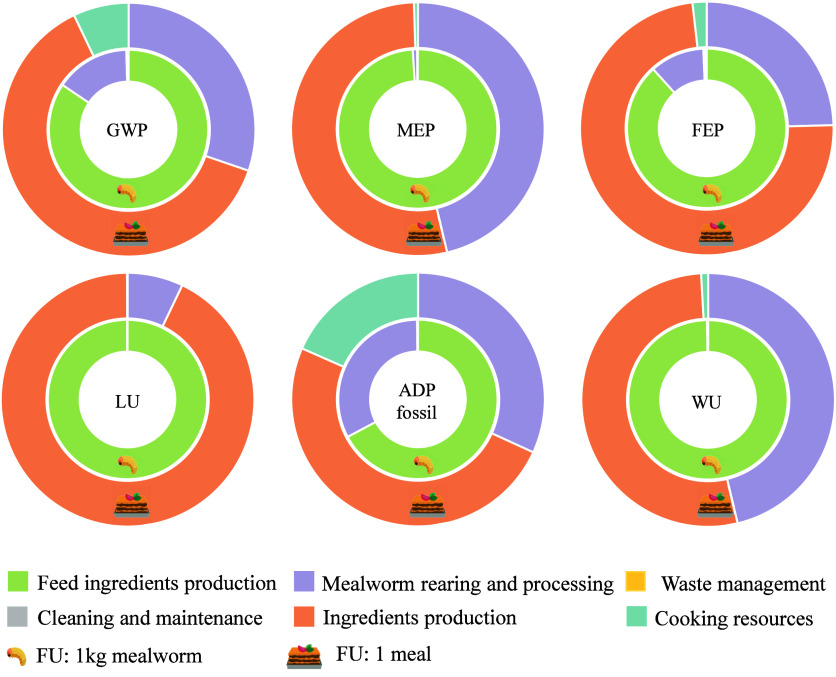
Relative contributions of the life cycle stages to the overall impacts of yellow mealworm production (inner circle) and meal production (outer circle).

The production and supply of ingredients for feed formulation for mealworm production were the largest contributors across all of the impact categories. This stage accounted for nearly 100% of the impacts in LU (99.9%), WU (99.9%), and MEP (99%), while its contribution was slightly lower for FEP (88.3%) and GWP (84.4%) and notably lower for ADP fossil (67.2%). This tendency was attributed to crops’ cultivation and the associated application of fertilizers and herbicides, intensive use of water resources for irrigation, and soil utilization. Additionally, the relatively high degree of mechanization in crop production impacted the utilization of fossil fuels and consequent carbon emissions. Mealworm production, including rearing, killing, and preserving, had an important role in ADP fossil (32.6%). To a lesser extent, it contributed to 15.1% to GWP and 11% to FEP. In all cases, burdens were primarily linked to the electricity consumed during freezing and blanching, while direct rearing emissions, the production and use of PP boxes, or the electricity consumption of the climatic chamber were considered trivial. Finally, the contributions from cleaning, facilities maintenance, and management of organic residues and wastewater were negligible across all indicators. These trends are similar to those reported by the other authors. Dreyer et al.^20^ agreed with this study that most of the impacts of the land use-related category came from feed ingredients production, while mealworm production had a notable influence on GWP, FEP, and ADP fossil. However, the contributions of this stage (≈50–77%) were higher than those obtained in the present study, which was a direct consequence of the large energy consumption for heating the rearing facility and the nonrenewable sources of the electricity mix. Thévenot et al.^18^ reported comparable relative impacts, highlighting the importance of the diet composition in MEP and LU, and the consumption of electricity for mealworm processing in cumulative energy demand.

With regard to the lasagna meal, the mealworm production presented quite variable contributions across indicators. The lowest contributions of this ingredient were estimated at 7.11% for LU, 24.6% for FEP, 30.3% for GWP, or 31.8% for ADP fossil, whereas they rose to 46.28% for MEP and 46.26% for WU. In general, the production of the other ingredients conforming to the meal constituted the principal driver of environmental degradation. They summed up 62.6% of the impacts of GWP, 53.2% of MEP, 73.6% of FEP, 92.8% of LU, 49.8% of ADP fossil, and 52.8% of WU. Cereals like flour or pasta, along with dairy products such as milk or cheese, accounted for more than half of the burdens. Vegetables also represented an important source of fossil energy consumption, while fats and other ingredients contributed notably to GWP and FEP. With relation to the cooking resources, their contribution was relatively modest, of 7.10% in GWP and 18.4% in ADP fossil, primarily due to the electricity consumption by the stove and oven.

### 3.2 Quantification of Environmental Impacts in the *Status Quo* (aLCA)

Figure 4 depicts the absolute environmental impacts obtained for each attributional LCA scenario (including those of the sensitivity analysis) and FU. Results on the uncertainty analysis are reported in the Supporting Information (Tables S.5 and S.6). In light of the graphs, it can be stated that the modeling approach had an important influence on the results. The same trend was observed across all impact categories: higher burdens were reported for yellow mealworm larvae and lasagna meal when economic allocation was applied (TM_AE_) compared to the mass-based allocation scenario (TM_AM_). In contrast, both frass and dried mealworm residue were attributed to greater impacts in TM_AM_ than in TM_AE_ due to the high weight of manure produced and relatively low market price. On the other hand, the largest environmental impacts for mealworm and lasagna were estimated in the TM_AW_ scenario, where all impacts were assigned to *Tenebrio* production, disregarding the cogeneration of frass and residues as valuable products. At this point, it is worth noting that in the TM_AM_ scenario, products and byproducts had the same impact up to the freezing stage. This is because a mass-proportional allocation criterion was used, in which the same life cycle processes are shared until the end of mealworm rearing. On this basis, it would be considered more appropriate to attribute 100% of the impacts to the mealworms or to employ an economic allocation, as this approach could not provide the most comprehensive results.

**Figure 4:**
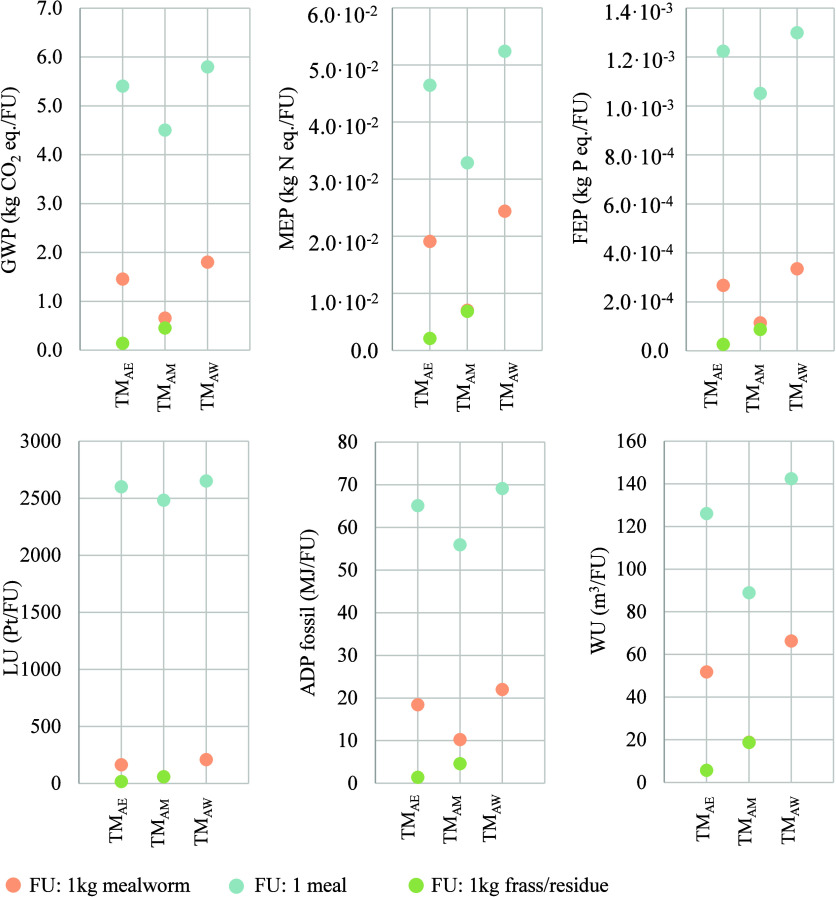
Environmental impacts of each product and byproduct considering different allocation scenarios: TM_AE_: base case or economic allocation; TM_AM_: mass-proportional allocation; and TM_AW_: 100% impacts allocated to mealworm production.

The production of 1 kg of yellow mealworm larvae resulted in a carbon footprint (CF) of 1.45 kg CO_2_ equiv when considering the base scenario (TM_AE_). Nevertheless, these emissions could vary between 0.66 (TM_AM_) and 1.80 (TM_AW_) kg CO_2_ equiv/kg depending on the modeling approach. Thévenot et al.^18^ calculated carbon emissions at 0.99 kg CO_2_ equiv/kg fresh larvae, attributing 100% of the impacts to mealworm production. On this basis and reducing system boundaries up to the rearing gate, an impact of 1.60 kg CO_2_ equiv/kg fresh larvae would be obtained in the present case study. A notable variance in the efficiency of production systems, i.e., the feed conversion ratios (1.98 vs 4), is the main reason for the difference in results. Similarly, Dreyer et al.^20^ reported a CF of 2.80 kg CO_2_ equiv/kg edible mealworm considering the whole production system. In this case, even though an FCR of 3.61 was estimated, the difference in results arose from the high energy demand for heating the rearing facility. Regarding the frass and mealworm residue, impacts between 0.14 (TM_AE_) and 0.45 kg (TM_AM_) CO_2_ equiv/kg were obtained, which cannot be compared with other reference values due to the lack of studies applying this type of allocation procedure. Finally, the production of mealworm lasagna generated 5.40 kg CO_2_ equiv in TM_AE_, 4.50 kg CO_2_ equiv in TM_AM_, and 5.79 kg CO_2_ equiv in TM_AW_. Lower variance was found in this scenario due to the relatively low contribution of *Tenebrio molitor* to the overall impacts.

Marine and freshwater eutrophication potentials accounted for 1.91 × 10^–2^ kg N equiv and 2.67 × 10^–4^ kg P equiv per kg of mealworm (TM_AE_) and could range from 7.03 × 10^–3^ (TM_AM_) to 2.44 × 10^–2^ (TM_AW_) kg N equiv and from 1.14 × 10^–4^ (TM_AM_) to 3.35 × 10^–4^ (TM_AW_) kg P equiv As a frame of comparison, FEP value for TM_AW_ was lower than that reported by Dreyer et al.,^20^ of 1.71 × 10^–3^ kg P equiv/kg, again as consequence of the high energy consumption during rearing. For the meal, emissions summed up to 1.13 × 10^–2^ kg N equiv and 7.94 × 10^–3^ kg P equiv per kg. In relation to resource consumption, land use was measured at 164.27 Pt/kg mealworm (TM_AE_), with a maximum value of 210.17 Pt in TM_AW_, while the water scarcity-related indicator reached 51.85 m^3^, with a maximum of 66.33 m^3^ in TM_AW_. Due to differences in the indicator’s coverage for each impact category, as well as the lack of studies, it was not possible to compare these values with other benchmarks. Land use impact for the production of the meal reached 2600.76 Pt/unit, mainly associated with the production of ingredients, and that of water scarcity accounted for 126.08 m^3^ (TM_AE_). Finally, fossil resources use ranged from 10.27 MJ (TM_AM_) to 22 MJ (TM_AW_), obtaining 18.42 MJ/kg of*Tenebrio molitor* larvae in the most realistic scenario (TM_AE_). The value obtained when all impacts are allocated to mealworms is slightly lower than that reported by Dreyer et al.^20^ (29.36 MJ), by the same reasoning as for other indicators. For the lasagna meal, similar energy consumptions were reported for all scenarios: 65.10 (TM_AE_), 55.92 (TM_AM_), and 69.12 MJ per meal (TM_AW_). Impacts for other FUs not mentioned in the text can be found in Figure 4.

### 3.3 Environmental Consequences of Changes in Demand (cLCA)

This section summarizes the main results of the consequential LCA modeling of the product system. Figure 5 shows the absolute impacts and relative contributions of each life cycle stage obtained for a growth in demand for *Tenebrio molitor* (FU: + 1 kg of mealworm) and for mealworm-based lasagna (FU: + 1 unit of meal).

**Figure 5:**
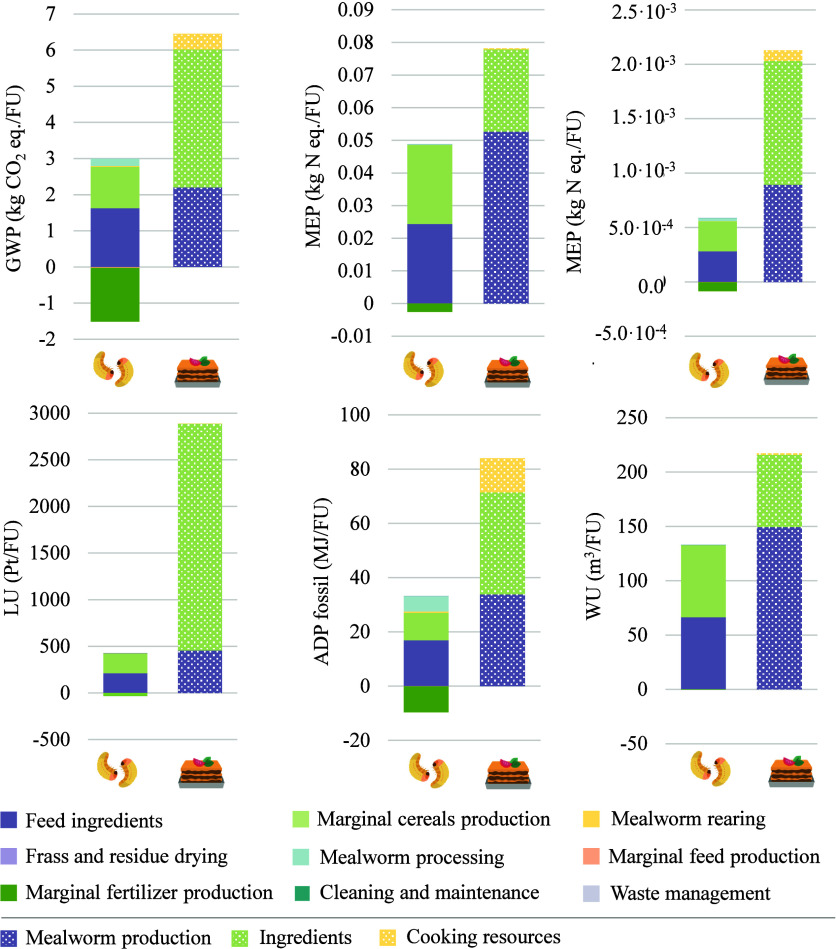
Absolute environmental impacts and relative contribution of each life cycle stage to the production of mealworm and lasagna meal under a cLCA approach.

Similar to the results from the aLCA, environmental impacts caused by the provision of feed ingredients for mealworm rearing were high in most of the impact categories. The consequential modeling identified the production of the additional cereal-based feed for insects as one of the most important contributors, ranging from 76.8 (GWP) to 43% (ADP fossil). The decrease in demand for commercial organic fertilizer production with insect frass resulted in positive environmental impacts or environmental credits. Major benefits were achieved in GWP by reducing impacts 100% or ADP fossil by 40.8%. The credit for using *Tenebrio molitor* as poultry feed was virtually negligible due to the small quantity of this byproduct. The inclusion of the marginal processes and system expansion gave rise to similar environmental burdens of *Tenebrio molitor* in comparison with the aLCA. Under a cLCA approach, the production of 1 additional kg of yellow mealworm larvae entailed a CF of 1.49 kg CO_2_ equiv, a fossil resources consumption 23.6 MJ, or a water scarcity potential of 132 m^3^. Impacts on other impact categories reached 4.62 × 10^–2^ kg N equiv, 5.01 × 10^–4^ kg P equiv, and 392 Pt per kg of product.

On the other hand, a growth in market demand for insect-based derivatives, specifically, mealworm-based lasagna, resulted in a CF of 5.25 kg of CO_2_ equiv/kg. Mealworm production entailed more than half of the impacts in MEP and WU, while other ingredients production did it in GWP, FEP, LU, and ADP fossil. Cooking resources only presented a meaningful contribution in the consumption of fossil resources as a consequence of the use of electricity and energy. In absolute values, impact in MEP reached 7.81 × 10^–2^ kg N equiv per additional meal, whereas that of FEP was estimated at 2.1210^–3^ kg P equiv. Burdens in LU, ADP fossil, and WU were calculated at 2887 Pt, 83.9 MJ, and 217 m^3^, respectively.

### 3.4 Comparison with Alternative Protein Sources: Nutritional and Environmental Perspectives

Results for the TM_AE_ scenario were used to compare the environmental impacts of *Tenebrio molitor* larvae and mealworm lasagna with other APs, considering mass-based and nutrient-based FUs (Figure 6). In this comparison, the water scarcity-related impact category was omitted due to the different food origins and the regionalization of the AWARE method, which could lead to confusing results. Additionally, it should be mentioned that for qNRF1.10.2 scores, some DIAAS were omitted since there is no available literature reporting these values for some ingredients or clinical trials for calculating the true ileal digestibility needed for the final estimation of the DIAAS.

**Figure 6:**
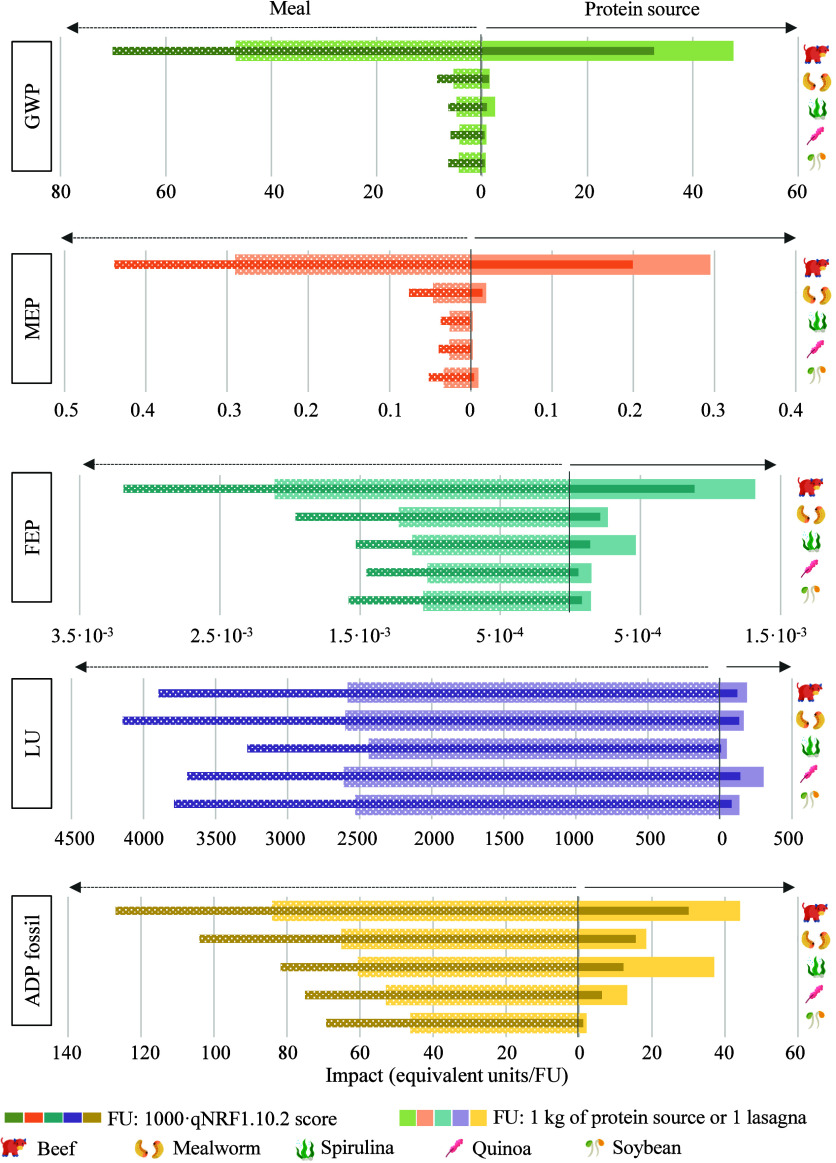
Environmental impacts of alternative protein sources and derived lasagnas considering mass- and nutrient-based FUs.

Beginning with the conventional approach, i.e., using a mass-based FU, the comparison of the environmental impacts of different protein sources allowed the identification of beef as the most detrimental product from the perspective of emissions generation. In terms of resource consumption, it led to the use of fossil resources, while quinoa led to land use. Trends for other foods were more variable, finding *Tenebrio molitor* in the middle of the ranking of most polluting foods. For instance, yellow mealworm reported a carbon footprint value between that of spirulina (2.48 kg CO_2_ equiv/kg) and quinoa (0.83 kg CO_2_ equiv kg), while it surpassed these products in terms of marine eutrophication. For its part, spirulina showed a notable impact in FEP (4.67 × 10^–4^ kg P equiv/kg) and ADP fossil (37 MJ/kg) due to the use of chemicals for culturing and the energy consumption for processing, but highlighted for its low soil occupation (46.9 Pt/kg). In general, soybean reported a similar environmental profile to that of quinoa, with most remarkable differences found in resource consumption (303 Pt/kg quinoa vs 133 Pt/kg soybean, or 13.2 MJ/kg quinoa vs 2.08 MJ/kg soybean). In relation to the meals, trends changed for most of the impact categories. Substitution of the protein sources was conducted considering the nutritional quality of the products, specifically by using the qNRF1.10.2 model. qNRF1.10.2 scores were estimated at 62.9 for mealworm, 204 for quinoa, 299 for spirulina, 146 for beef, and 151 for soybean. The wide variation between the scores’ values influenced the amount of ingredient to be added to the recipe to replace meat (original meal), which in turn influenced the environmental results. Consequently, mealworm-based lasagna generally showed one of the worst environmental performances due to the high amount of *Tenebrio* needed. Beef lasagna accounted for 46.7 kg CO_2_ equiv/unit, while that of mealworm decreased to 5.40 kg CO_2_ equiv/unit, although it was still higher than that of quinoa, soybean or spirulina. The same tendency was observed for MEP, FEP, and ADP fossil, while for LU, quinoa and mealworm lasagnas led the ranking.

On the other hand, the adoption of a nutritional LCA perspective did not lead to strong changes in the results’ trends. The integration of the nutritional quality into the environmental performance of *Tenebrio* resulted in a CF of 1.24 kg CO_2_ equiv, a land use impact of 141 Pt, a fossil resources consumption of 15.7 MJ, and impacts on eutrophication of 1.63 × 10^–2^ kg N equiv and 2.28 × 10^–4^ kg P equiv per FU of 1000 × qNRF1.10.2 score. When compared to alternative protein sources, tendencies were similar to those reported for the mass-based FU, with major differences arising from the spirulina profile. The high nutritional quality of this product made it more competitive in terms of carbon emission (0.83 kg CO_2_ equiv/1000 × qNRF1.10.2 score), freshwater eutrophication (1.56 × 10^–4^ kg P equiv), and ADP fossil (37 MJ), which were previously identified as critical. Even though the inclusion of the nutritional properties of beef brought it closer to other products in terms of environmental impact, these were still far superior. Regarding soybean and quinoa, no significant changes were observed. Turning to the protein-rich meals, the increase in the burdens as a consequence of the introduction of nutritional factors in the FU is noteworthy. qNRF1.10.2 scores were calculated at 67 for soybean lasagna, 70.8 for quinoa lasagna, 74.5 for spirulina lasagna, 62.9 for *Tenebrio* lasagna, and 66.5 for beef lasagna. It is important to understand the differences between these values: even though the protein source replacement was performed to ensure that the alternative protein provides the same nutritional quality as the initial one, the weight of the ingredient varies, and with it, the weight of the final product and the score. Overall and again, trends were similar to those reported for the mass-based FU. As in the conventional evaluation, mealworm lasagna did not report a promising environmental profile, additionally driven by the low nutritional score: it was the second most polluting product, following beef, in GWP (8.58 kg CO_2_ equiv/1000 × qNRF1.10.2 score), FEP (1.90 × 10^–3^ kg P equiv), MEP (3.74 × 10^–2^ kg N equiv), and ADP fossil (103 MJ), and first in land occupation (4133 Pt). The only difference was reported for the land use of quinoa and soybean, whose trend was reversed due to the nutritional value of the former.

## 4 Conclusions

In light of the exerted pressure of protein-rich products on the environmental implications of food systems and their upcoming demand, this study aims to evaluate the environmental performance of *Tenebrio molitor* as a novel nutrient source. This was done by applying various LCA methodological approaches: (i) proposal of three attributional models with different allocation strategies, (ii) study of a consequential model to assess the influence of a growth in demand for mealworm-based products, and (iii) consideration of a nutritional LCA approach to take into account the nutritional function of the product in the environmental performance.

Results evidenced a strong influence as a consequence of the modeling approach: higher environmental burdens were reported for the larvae and the mealworm lasagna when no allocation was considered (TM_AW_), followed by the economic allocation scenario (TM_AE_), and finally the mass-allocation model (TM_AM_). For the baseline case (TM_AE_), a carbon footprint of 1.45 kg CO_2_ equiv, a land use of 164 Pt, and a fossil resources consumption of 18.4 MJ per kg of yellow mealworm larvae were reported, for which the major contributor was the feed ingredients production. For the meal, impacts of 5.40 kg of CO_2_ equivalent, 2601 Pt, or 65.1 MJ per lasagna were estimated. The consequential model identified the substitution of commercial fertilizer with mealworm frass as the main environmental benefit, while the marginal production of cereals entailed important additional impacts. For the lasagna system, mealworm and other ingredients production were the main drivers of impacts, and the carbon footprint was estimated at 5.25 kg CO_2_ equiv/meal. Regarding the nutritional LCA perspective, the integration of qNRF1.10.2 into the environmental assessment indicated a great potential of yellow mealworm larvae with respect to other animal products, in particular beef, mainly due to the low direct emissions of the rearing and the high feed conversion ratio. However, in comparison with plant-based alternatives, such as quinoa, soybean, or spirulina, the environmental profile of *Tenebrio molitor* is not such competitive, which was associated with the feed consumption from the insect but also with its relatively low nutritional quality (117 vs 299 of spirulina or 204 of quinoa).

Overall, this study provides a valuable contribution to the future of the insect industry, highlighting the hotspots and bottlenecks of the productive system and setting directions for potential improvements. One key limitation of this study concerns the adoption of a consequential approach. For the modeling of the lasagna system, the Agribalyse database, which follows an attributional logic, was applied. This may reveal an inconsistency between the study’s goal and its methodology, and, although for this system the influence may be low or minimal, it could lead to uncertainty as a consequence of relevant differences in environmental outcomes between attributional and consequential processes.^37^ Consequently, further research is needed in this field to obtain the most reliable outcomes that are possible. On the other hand, future research should explore the implications of indirect land use change (iLUC), particularly in relation to feed production and land competition. As iLUC can significantly affect the overall environmental performance of food systems, incorporating robust and context-specific iLUC modeling would provide a more comprehensive understanding of the long-term sustainability of insect-based products.^38^

From a practical perspective, upscaling the production may have a direct implication on its environmental profile by improving the feed conversion and energy usage. Given that the main hotspot is not the insect rearing itself, but the upstream processes, the valorization of agricultural residues or livestock feed waste for insect production could present some initial technical challenges but become a feasible alternative for enhancing environmental efficiency in the medium term. Rearing temperature is another key external factor that affects metabolism and growth rate and has a strong impact on the optimization of production time, efficiency, and final quality. For that reason, this parameter should be exhaustively controlled in edible insects’ industrialization by the design and application of energy-efficient environment-control equipment that optimizes the production. However, despite these opportunities, entomophagy also presents challenges. One of the main handicaps resides in the consumers’ acceptance and willingness to pay for and eat insect-derived products. The social acceptability of reared insects is variable and impacted by diverse factors, including geographic location, legislation, gender, and socio-economic status. In European markets, it is still a nascent market segment, and many citizens are reluctant to introduce these products into their diets. The need to “camouflage” insects among other ingredients or to mimic the taste, texture, or smell of other animal products is still one of the initial challenges to address this social issue and promote the development of this sector.

## Supplementary Material



## Notes

Background data and adaptations of the background processes for modeling the attributional and consequential production systems; environmental impacts of the yellow mealworm and lasagna production systems (FU: 1 kg), with medians, standard deviations and fifth and 95th percentiles of the probability distribution functions based on Monte Carlo iterations; environmental impacts on other indicators for the attributional scenario with economic allocation and consequential approach (PDF)
