# Supplementary material for: Environmental Evaluation of Yellow Mealworm Larvae Products: Analysis of Modeling Choices and Nutritional Impact-Adjusted Comparison
Source: ACS Omega. 2026 Feb 6;11(6):8963–75. doi: 10.1021/acsomega.5c03159 (PMC12917628; doi:10.1021/acsomega.5c03159)
Supplement: Supplementary file 1 [file ao5c03159_si_001.pdf]

Supporting Information - Environmental evaluation of yellow mealworm larvae products: analysis of modeling choices and nutritional impact-adjusted comparison

Ana Fernández-Ríos[1,*], Jara Laso[1], Rubén Aldaco[1], María Margallo[1]

[1]*Department of Chemical and Biomolecular Engineering, University of Cantabria. Av. de los Castros s/n, 39005 Santander, Spain.*

Table S. 1: Background data and adaptations of the background processes for modeling the attributional production systems.

Resource	Root process	Modifications
Wheat bran	Wheat grain {ES}|wheat grain production|Cut-off, U	Grain milling and bran obtention data from Câmara-Salim et al.[1]: addition of pyrethrin 0.2% for pollution control during storage, and water and electricity from Spanish grid mix for milling
Oat	Oat grain {FI}|oat grain production|Cut-off, U	Adaptation of oat production under Finnish conditions: change of background processes for regionalization {ES}, {RER} or {Europe without Switzerland} whenever possible
Corn	Maize grain {IN}|maize grain production|Cut-off, U	Adaptation of maize production in India according to specifications of Abrahão et al.[2]: change of background processes for regionalization {ES}, {RER} or {Europe without Switzerland}
Soybean	Soybean {CH}|soybean production|Cut-off, U	Adaptation of soybean production in Switzerland: change of background processes for regionalization {ES}, {RER} or {Europe without Switzerland}
Potato	-	Life cycle inventory extracted from Fernández-Ríos et al. [3]
Barley	Barley grain {ES}|barley grain production|Cut-off, U	None
Land use	Transformation, to industrial area, ES	None
Transportation	Transport, freight, lorry 3.5-7.5 metric ton, EURO6 {RER}| market for transport, freight, lorry 3.5-7.5 metric ton, EURO6 | Cut-off, U	None
PP (packaging)	Polypropylene, granulate {RER}| polypropylene production, granulate | Cut-off, U	None

	Injection moulding {RER}| injection moulding | Cut-off, U	
Tap water	Tap water {Europe without Switzerland}| market for tap water | Cut-off, U	None
Bleach	Bleach {RER}| sodium hypochlorite to generic market for bleach | Cut-off, U	None
Anti-mite solution	Pyrethroid-compound {RER}| pyrethroid-compound production | Cut-off, U<br>Rape oil, crude {RoW}| market for rape oil, crude | Cut-off, U	Modeled following composition of commercial product: 4.59 g/L of pyrethrin and 823.3 g/L of rapeseed oil
Mop (soil cleaner)	Non-ionic surfactant {GLO}| non-ionic surfactant production, ethylene oxide derivate | Cut-off, U	Modeled following composition of commercial products: 5% concentration of non-ionic surfactant products
	Water, deionised {Europe without Switzerland}| market for water, deionised | Cut-off, U	None
Wastewater treatment	Wastewater, average {Europe without Switzerland}| treatment of wastewater, average, wastewater treatment | Cut-off, U	None
Organic waste management in Spain	Biowaste {CH}| treatment of biowaste, industrial composting | Cut-off, U<br>Municipal solid waste {RoW}| treatment of municipal solid waste, sanitary landfill | Cut-off, U<br>Municipal solid waste {ES}| treatment of municipal solid waste, municipal incineration | Cut-off, U	Modeled based on real data, which assume 5.67% of the organic waste is recycled, 22.90% composted, 58.15% landfilled and 13.28% incinerated
Residual electricity mix	-	Modeled based on the residual electricity mix composition reported in AIB (2024): 58% fossil resources, mainly natural gas and coal, 36% nuclear, and 6% renewable energy, especially wind and solar. Low voltage electricity was assumed.
Olive oil	Extra Virgin Olive Oil, at plant {ES} U	Plastic packaging included according to references
Onion	Onion, cooked, processed in FR | Ambient (average) | PP | at packaging {FR} U	None

Garlic	Garlic, fresh, processed in FR | Ambient (long) | No packaging | at packaging {FR} U	None
Wheat	Wheat grain {ES}| wheat grain production | Cut-off, U	None
Beef stock	Broth, stock or bouillon, beef, processed in FR | Ambient (long) | Corrugated board and aluminium packaging | at packaging {FR} U	None
Tomato sauce	Tomato, fresh grade {ES}| tomato production, fresh grade, in unheated greenhouse | Cut-off, U	Additional resources included to produce tomato sauce. Reference from Agribalyse database
Thyme	Thyme, dried, processed in FR | Ambient (long) | Glass | at packaging {FR} U	None
Tomato	Tomato, fresh grade {ES}| tomato production, fresh grade, in unheated greenhouse | Cut-off, U	None
Wheat flour	Wheat grain {ES}| wheat grain production | Cut-off, U	Milling operation and packaging considered based on Agribalyse database for flours
Milk	Cow milk {ES}| milk production, from cow | Cut-off, U	None
Mustard	Mustard, processed in FR | Ambient (long) | Glass | at packaging {FR} U	None
Parmesan cheese	Parmesan cheese, from cow's milk, processed in FR | Chilled | LDPE | at packaging {FR} U	Adaptation to milk produced in Spain
Dried pasta	Dried pasta, raw, processed in ES | Ambient (long) | LDPE | at packaging {FR} U	None
Parmesan cheese	Parmesan cheese production, from cow's milk, hard cheese, French production mix, at plant, 1 kg of Parmesan cheese (PGi) {FR} U	Adaptation to milk produced in Spain

Table S. 2: Background data and adaptations of the background processes for modeling the consequential production systems. Processes for the lasagna system were the same as for the attributional model.

**Resource**	**Root process**	**Modifications**
Residual electricity mix	-	Modeled based on the residual electricity mix composition reported in AIB (2024): 58% fossil resources, mainly natural gas and coal, 36% nuclear, and 6% renewable energy, especially wind

		and solar. Root processes taken from the consequential model.
Organic fertilizer	Organic fertiliser, 3-2-3, bulk {RER} U	None
Black soldier fly	Black soldier fly fresh larvae, at processing {FR} U	None
Wheat bran	Wheat grain {ES}|wheat production|Conseq, U	Grain milling and bran obtention data from Câmara-Salim et al.[1]: addition of pyrethrin 0.2% for pollution control during storage, and water and electricity from Spanish grid mix for milling. Root processes taken from the consequential model.
Oat	Oat grain {FI}|oat production|Conseq, U	Adaptation of oat production under Finnish conditions: change of background processes for regionalization {ES}, {RER} or {Europe without Switzerland} whenever possible. Root processes taken from the consequential model.
Corn	Maize grain {IN}|maize production|Conseq, U	Adaptation of maize production in India according to specifications of Abrahão et al.[2]: change of background processes for regionalization {ES}, {RER} or {Europe without Switzerland}. Root processes taken from the consequential model.
Soybean	Soybean {CH}|soybean production|Conseq, U	Adaptation of soybean production in Switzerland: change of background processes for regionalization {ES}, {RER} or {Europe without Switzerland}. Root processes taken from the consequential model.
Potato	-	Life cycle inventory extracted from Fernández-Ríos et al. [3] Root processes taken from the consequential model.
Barley	Barley grain {ES}|barley production|Conseq, U	None
Transportation	Transport, freight, lorry 3.5-7.5 metric ton, euro6 {RER}| market for transport, freight, lorry 3.5-7.5 metric ton, EURO6 | Conseq, U	None
PP (packaging)	Polypropylene, granulate {RER}| production | Conseq, U	None

	Injection moulding {RER}| processing | Conseq, U	
Tap water	Tap water {Europe without Switzerland}| market for | Conseq, U	None
Bleach	Bleach {RER}| sodium hypochlorite to generic market for bleach | Cut-off, U	None
Anti-mite solution	Pyrethroid-compound {RER}| production | Conseq, U<br>Rape oil, crude {CH}| market for | Conseq, U	Modeled following composition of commercial product: 4.59 g/L of pyrethrin and 823.3 g/L of rapeseed oil
Mop (soil cleaner)	Non-ionic surfactant {GLO}| non-ionic surfactant production, ethylene oxide derivate | Conseq, U	Modeled following composition of commercial products: 5% concentration of non-ionic surfactant products
	Water, deionised {Europe without Switzerland}| market for water, deionised | Conseq, U	None
Wastewater treatment	Wastewater, average {Europe without Switzerland}| treatment of wastewater, average, capacity 1E9l/year | Conseq, U	None
Organic waste management in Spain	Biowaste {CH}| treatment of biowaste, industrial composting | Conseq, U<br>Municipal solid waste {RoW}| treatment of municipal solid waste, sanitary landfill | Conseq, U<br>Municipal solid waste {ES}| treatment of municipal solid waste, municipal incineration | Conseq, U	Modeled based on real data, which assume 5.67% of the organic waste is recycled, 22.90% composted, 58.15% landfilled and 13.28% incinerated

Table S. 3: Environmental impacts on other impact categories for the TM_{AE} scenario.

**Impact category**	**FU: 1 kg mealworm**	**FU: 1 kg frass**	**FU: 1 kg residue**	**FU: 1 meal**
Acidification [mol H+ eq.]	$4.70\cdot10^{-2}$	$5.19\cdot10^{-3}$	$5.19\cdot10^{-3}$	0.11
Ecotoxicity, freshwater (inorganics) [CTUe]	6.66	0.72	0.72	23.11
Ecotoxicity, freshwater (organics) [CTUe]	9.78	1.04	1.04	60.38
Particulate matter [disease inc.]	$3.29\cdot10^{-7}$	$3.62\cdot10^{-8}$	$3.62\cdot10^{-8}$	$7.37\cdot10^{-7}$

Eutrophication, terrestrial [mol N eq.]	0.21	$2.30\cdot10^{-2}$	$2.30\cdot10^{-2}$	0.45
Human toxicity, cancer [CTUh]	$8.86\cdot10^{-10}$	$9.47\cdot10^{-11}$	$9.47\cdot10^{-11}$	$3.30\cdot10^{-9}$
Human toxicity, non-cancer [CTUh]	$7.82\cdot10^{-8}$	$8.56\cdot10^{-9}$	$8.56\cdot10^{-9}$	$2.11\cdot10^{-7}$
Ionising radiation [kBq U-235 eq.]	0.22	$6.05\cdot10^{-3}$	$6.05\cdot10^{-3}$	0.92
Ozone depletion [kg CFC11 eq.]	$2.67\cdot10^{-8}$	$2.61\cdot10^{-9}$	$2.61\cdot10^{-9}$	$1.70\cdot10^{-7}$
Photochemical ozone formation [kg NMVOC eq.]	$6.18\cdot10^{-3}$	$6.14\cdot10^{-4}$	$6.14\cdot10^{-4}$	$1.92\cdot10^{-2}$
Resource use, minerals and metals [kg Sb eq.]	$1.23\cdot10^{-6}$	$1.35\cdot10^{-7}$	$1.35\cdot10^{-7}$	$9.62\cdot10^{-6}$

The results for the additional impact categories in the TM_{AE} scenario (Table S.3) were consistent with the main findings reported in the manuscript. In most categories, ingredients provision, including production and transportation, entailed the greatest environmental impacts, as consequence of the use of fertilizers, pesticides, and other materials that have an important influence on resources consumption or toxicity- and ecotoxicity-related indicators. Consistently, burdens were higher for mealworm production than for frass and residue. For instance, for this former, impacts were estimated at $4.70\cdot10^{-2}$ mol H+ eq. in acidification, 0.22 kBq U-235 eq. in ionizing radiation, or $1.23\cdot10^{-6}$ kg Sb eq. in natural resources consumption. With respect to the lasagna meal, ingredients production still presented the main driver of impacts in most additional categories, which reached 0.45 mol N eq. in terrestrial eutrophication, $1.70\cdot10^{-7}$ kg CFC11 eq. in ozone depletion, or $9.62\cdot10^{-6}$ in resources use.

Table S. 4: Environmental impacts on other impact categories for the TM_C scenario.

**Impact category**	**FU: 1 kg mealworm**	**FU: 1 meal**
Acidification [mol H+ eq.]	0.11	0.18
Ecotoxicity, freshwater (inorganics) [CTUe]	46.46	77.4
Ecotoxicity, freshwater (organics) [CTUe]	18.46	38.4
Particulate matter [disease inc.]	$6.76\cdot10^{-7}$	$1.22\cdot10^{-6}$
Eutrophication, terrestrial [mol N eq.]	0.46	0.74
Human toxicity, cancer [CTUh]	$3.34\cdot10^{-9}$	$7.64\cdot10^{-9}$
Human toxicity, non-cancer [CTUh]	$1.94\cdot10^{-7}$	$3.94\cdot10^{-7}$
Ionizing radiation [kBq U-235 eq.]	0.10	0.83
Ozone depletion [kg CFC11 eq.]	$2.80\cdot10^{-7}$	$6.54\cdot10^{-7}$
Photochemical ozone formation [kg NMVOC eq.]	$9.98\cdot10^{-3}$	$2.86\cdot10^{-2}$
Resource use, minerals and metals [kg Sb eq.]	$1.40\cdot10^{-5}$	$1.64\cdot10^{-5}$

Following a similar trend than that reported for the main impact categories analyzed, marginal cereals production was one of the main contributors to the total burdens in mealworm production, while the decrease in the demand for organic fertilizers was associated with the main environmental credit. With regard to lasagna, mealworm and other ingredients production resulted to be the main hotspots in most additional categories, whereas the cooking resources had, generally, a negligible contribution. In terms of values, impacts on acidification were calculated at 0.11 and 0.18 mol H+ eq. per kg of mealworm and per one meal, respectively, while those of terrestrial eutrophication were measured at 0.46 and 0.74 mol N eq. Impacts on other impact categories can be consulted in Table S.4.

Table S. 5: Environmental impacts of the yellow mealworm production system (FU: 1 kg). Medians, standard deviations and 5th and 95th percentiles of the probability distribution functions based on Monte Carlo iterations are reported.

Impact category	Median	SD	2.5^{th} percentile	97.5^{th} percentile
Scenario TM_{AE}				
GWP [kg CO_2 eq.]	1.45	$9.42 \cdot 10^{-2}$	1.28	1.65
FEP [kg P eq.]	$2.59 \cdot 10^{-4}$	$4.76 \cdot 10^{-5}$	$2 \cdot 10^{-4}$	$3.75 \cdot 10^{-4}$
MEP [kg N eq.]	$1.87 \cdot 10^{-2}$	$2.84 \cdot 10^{-3}$	$1.43 \cdot 10^{-2}$	$2.61 \cdot 10^{-2}$
LU [Pt]	185	37*	-566	942
ADP fossil [MJ]	18.3	2.01	15	23
WU [m^3]	52.8	26	-2.33	101
Scenario TM_{AM}				
GWP [kg CO_2 eq.]	0.65	$3.86 \cdot 10^{-2}$	0.57	0.73
FEP [kg P eq.]	$1.08 \cdot 10^{-4}$	$2.91 \cdot 10^{-5}$	$7.91 \cdot 10^{-5}$	$1.95 \cdot 10^{-4}$
MEP [kg N eq.]	$6.91 \cdot 10^{-3}$	$1.01 \cdot 10^{-3}$	$5.27 \cdot 10^{-3}$	$9.27 \cdot 10^{-3}$
LU [Pt]	77.8	139	-206	362
ADP fossil [MJ]	10.1	1.32	8.05	13.2
WU [m^3]	19.6	9.47	-1.36	38.2
Scenario TM_{AW}				
GWP [kg CO_2 eq.]	1.80	0.12	1.57	2.05
FEP [kg P eq.]	$3.27 \cdot 10^{-4}$	$5.66 \cdot 10^{-5}$	$2.56 \cdot 10^{-4}$	$4.64 \cdot 10^{-4}$
MEP [kg N eq.]	$2.42 \cdot 10^{-2}$	$4.01 \cdot 10^{-3}$	$1.87 \cdot 10^{-2}$	$3.40 \cdot 10^{-2}$
LU [Pt]	213	480	-705	1170
ADP fossil [MJ]	21.6	2.57	17.8	28.4
WU [m^3]	67.8	34.5	-9.55	130.1

Table S. 6: Environmental impacts of the mealworm-based lasagna production system (FU: 1 meal). Medians, standard deviations and 5th and 95th percentiles of the probability distribution functions based on Monte Carlo iterations are reported.

Impact category	Median	SD	2.5th percentile	97.5th percentile
Scenario TM_{AE}				
GWP [kg CO_2 eq.]	6.61	3.48	-0.37	13.61
FEP [kg P eq.]	$1.46\cdot10^{-3}$	$1.98\cdot10^{-4}$	$1.21\cdot10^{-3}$	$1.99\cdot10^{-3}$
MEP [kg N eq.]	$6.74\cdot10^{-2}$	$9.67\cdot10^{-3}$	$5.16\cdot10^{-2}$	$9.04\cdot10^{-2}$
LU [Pt]	2632	1562	-320	5941
ADP fossil [MJ]	80.1	7.43	68.6	99.3
WU [m^3]	293	1109	-2113	2176
Scenario TM_{AM}				
GWP [kg CO_2 eq.]	5.94	3.30	-1.03	12.3
FEP [kg P eq.]	$1.29\cdot10^{-3}$	$1.56\cdot10^{-4}$	$1.08\cdot10^{-3}$	$1.65\cdot10^{-3}$
MEP [kg N eq.]	$5.35\cdot10^{-2}$	$7.54\cdot10^{-3}$	$4.26\cdot10^{-2}$	$7.06\cdot10^{-2}$
LU [Pt]	2635	1215	196	5088
ADP fossil [MJ]	69.5	5.97	60.1	82.9
WU [m^3]	243	1090	-2284	2244
Scenario TM_{AW}				
GWP [kg CO_2 eq.]	6.98	3.68	-0.45	14.6
FEP [kg P eq.]	$1.54\cdot10^{-3}$	$1.72\cdot10^{-4}$	$1.26\cdot10^{-3}$	$1.93\cdot10^{-3}$
MEP [kg N eq.]	$7.18\cdot10^{-2}$	$9.39\cdot10^{-3}$	$5.70\cdot10^{-2}$	$9.24\cdot10^{-2}$
LU [Pt]	2978	1620	-301	6012
ADP fossil [MJ]	83.2	7.04	70.3	100
WU [m^3]	342	1133	-2354	2230

The uncertainty evaluation performed through the Monte Carlo analysis demonstrated that uncertainty levels varied across the scenarios of aLCA. The baseline case, i.e., TM_{AE}, reported the lowest uncertainty factors in ADP fossil and WU, while TM_{AM} did it for GWP, FEP and LU. Only MEP showed the minimum uncertainty values in the scenario with no allocation (TM_{AW}). For the production of *Tenebrio molitor*, relatively low standard deviation, expressed as the percentage of deviation from the average value, was observed for climate change, which ranged from 5.93% to 6.56%, ADP fossil (10.9%-13%), MEP (14.5%-16.1%) and FEP (16.9%-25.5%). In contrast, higher uncertainty was indicated for WU, with a maximum of 51.3%, and LU, of 221%, both obtained in TM_{AW}. This high variability may be associated with the use of the background dataset, in particular with those processes linked to the production of feed ingredients that had the greatest impact on these two categories, along with the inherent uncertainty of the impact methods. For the production of the meal, the lowest uncertainty levels were reported for TM_{AW}. An exception was found for LU, for which the TM_{AM} scenario showed the minimum deviation. As for the mealworm production system, relatively low standard

deviations were estimated for ADP fossil (8.43%-9.20%), FEP (11.1%-13.3%) and MEP (13%-14.2%). However, deviations up to 50% were indicated in this case for GWP, with a maximum of 56.4% obtained in TM_{AM}, and LU, with a maximum of 57.2%. Finally, the water scarcity-related category reported the highest uncertainty (511%-561%), which may be linked with the production of feed ingredients and the regionalization of the production.

References

(1) Câmara-Salim, I., Almeida-García, F., González-García, S., Romero-Rodríguez, A., Ruiz-Nogueiras, B., Pereira-Lorenzo, S., Feijoo, G., Moreira, M.T. Life cycle assessment of autochthonous varieties of wheat and artisanal bread production in Galicia, Spain. *Sci. Total Environ.* **2020**, *713*, 136720. https://doi.org/10.1016/j.scitotenv.2020.136720

(2) Abrahão, R., Carvalho, M., Causapé, J. Carbon and water footprints of irrigated corn and non-irrigated wheat in Northeast Spain. *Environ. Sci. Pollut. Res*. **2017**, *24,* 5647-5653. https://doi.org/10.1007/s11356-016-8322-5

(3) Fernández-Ríos, A., Laso, J., Amo-Setién, F.J., Abajas-Bustillo, R., Ortego-Maté, C., Fullana-i-Palmer, P., Bala, A., Batlle-Bayer, L., Balcells, M., Puig, R., Aldaco, R., Margallo, M. Water-energy-food nexus and life cycle thinking: a new approach to environmental and nutritional assessment of potato chips. *Foods* **2022,** *11*, 1018. https://doi.org/10.3390/foods11071018
